# Evolutionary perspectives on human behavior during the coronavirus pandemic: Insights from game theory

**DOI:** 10.1093/emph/eoaa034

**Published:** 2020-09-07

**Authors:** Martin Brüne, Daniel R Wilson

**Affiliations:** e1 Division of Social Neuropsychiatry and Evolutionary Medicine, Department of Psychiatry, Psychotherapy and Preventive Medicine, LWL University Hospital, Ruhr-University, Bochum, Germany; e2 President, Western University of Health Sciences, Pomona, CA, USA

**Keywords:** coronavirus, behavioral immune system, sickness behavior, game theory, public goods game

## Abstract

The coronavirus pandemic constitutes a global challenge to society and medicine. Here, we review evolutionary insights that are relevant for the understanding of how people respond to the pandemic and what to expect in the aftermath of the crisis. Specifically, we argue that the behavioral immune system (BIS) and sickness behavior (SB) comprise two adaptive responses to impending and actual infection, respectively, and that individuals activating their BIS differ from those showing SB in important ways that may have implications for the prevention and treatment of COVID-19. Moreover, we reframe some of the behavioral health issues associated with the pandemic in a game-theoretical scenario, illustrating the difficulties that arise when public health is treated as a ‘public good’.

Lay summary: The coronavirus pandemic constitutes a global challenge to society and medicine. In this article, we employ evolutionary theory to improve our understanding of how people respond to the pandemic. Specifically, we argue that human behavior is guided by ancient mechanisms involving either the avoidance of infection or defense against attacks in times of enhanced vulnerability. Moreover, we reframe some of the behavioral health issues associated with the pandemic in a game-theoretical scenario. This helps understand why most people comply with rules of social distancing, while a minority fails to do so for very different reasons. The evolutionary perspective also allows making some predictions for the course of the pandemic.

## INTRODUCTION

The current pandemic caused by coronavirus SARS-CoV-2 is an extraordinary global challenge to society and medicine. What began as a medical crisis has rapidly and directly now led to crises of a social, economic and even existential nature. Healthcare systems, even in developed countries, are overburdened by increasing numbers of critically ill patients, and in some places at risk of collapse. Here, we consider how evolutionary perspectives on health issues associated with the pandemic can explain difficulties in handling infection risk and in dealing with at-risk populations and manifest COVID-19 patients. This viewpoint is consistent with previous work, suggesting that evolutionary insights are mandatory in infectious disease surveillance, among other topics of interest to public health [1]. Specifically, we argue that the behavioral immune system (BIS) and sickness behavior (SB) are important adaptive responses to impending infection and actual infection, respectively, which may be associated with disparate behavioral attitudes impacting prevention and treatment of COVID-19. The problem of how to deal with the crisis, we assert, can be reframed in a game-theoretical scenario, such as the ‘Public Goods Game’, whereby predictable uncertainties arise from conceptual difficulties in defining public health as a ‘public good’.

## INFECTIOUS DISEASES AND HUMAN EVOLUTION

Infectious disease has clearly shaped the human immune system for most of our evolutionary history. It is less widely appreciated, however, that infectious disease has also shaped human behavior, though it has become increasingly obvious how tightly the immune system is mutually linked to brain function [2]. Humans evolved defense mechanisms against infectious diseases, known as the ‘behavioral immune system’ (BIS) [3]. This system is, by no means, specific to humans; even insects and lobsters avoid contact with infected conspecifics [4, 5], and in higher vertebrates, this avoidance seems to be mediated by the emotion of disgust [6], which is closely linked to the physical immune system [7]. The human BIS comprises the avoidance of social interactions posing a potential infection risk, conformity and maintenance of cultural norms, i.e. ingroup coherence, as well as neophobia. Activation of the BIS is thus associated with heightened vigilance toward and avoidance of outgroup members, with linkages to the fear system [8]. Indeed, it has been shown that humans are able to recognize even subtle signs of sickness in others, which activates an immune response in the observer, and fosters rejection and avoidance of the sick individual [3].

While the BIS is activated by healthy individuals to prevent infection, ‘sickness behavior’ (SB) refers to a psychophysiological state usually caused by infectious agents (though in modern environments, it can also occur in autoimmune disease, allergies and immune-modulating drug treatment). SB is characterized by fatigue, loss of appetite and drive, psychomotor retardation and social withdrawal. SB is also frequently associated with loss of appetite, which reduces the exposure to toxic or infectious material, and heightened body temperature. As such, it reflects an adaptive evolutionarily conserved defense reaction to conserve energy and reduce the risk of being attacked in times of enhanced vulnerability [9]. Some researchers have therefore highlighted the similarities between SB and clinical depression, including immunological theories suggesting that aberrant priming of the immune system could be part of an explanation for the ‘depression pandemic’, because the divergent exposure to certain pathogens in modern environments compared to ancestral ones has created an evolutionary ‘mismatch’ [10–12]. Similar to people activating their BIS, individuals displaying SB avoid contact with strangers (for different reasons, however), but seek proximity to close kin, even though this may impose costs to inclusive fitness, because it increases the risk of infection of genetically related individuals [3].

With regard to the SARS-CoV-2 pandemic, aside from individuals showing signs of BIS or SB, at-risk groups deserve special medical attention. They comprise a heterogeneous array of syndromal or diagnostic categories, including people with pulmonary or cardiovascular disease, obesity, metabolic syndrome and diseases associated with compromised immune function. Accordingly, age is also considered a risk-factor for severe or fatal courses of COVID-19 [13]. In addition, many with psychiatric conditions are burdened with one or more comorbid disorders listed above, which makes them vulnerable to poor outcome of COVID-19, too. There is also evidence to suggest that socially under-privileged persons suffer more from the pandemic than economically more affluent individuals [14]. With respect to public health issues in relation to the SARS-CoV-2 pandemic, populations comprising a relatively large number of unaffected individuals, an increasing number of infected people and a sizeable proportion of at-risk subjects with regard to outcome, may act in quite specific and partly predictable ways that may require public health measures and intervention programs. The at-risk group is relevant from an evolutionary point of view, specifically, because one can expect BIS and SB to occur more frequently in these patients compared with the general population, and some (especially those with psychiatric disorders, and the elderly) may be most affected from the costs of social distancing, including social isolation, and loss of key benefits of sociality, such as within-group cooperation and provision of safety [summarized in 15].

## A GAME-THEORETICAL APPROACH TO THE SARS-CoV-2 PANDEMIC

The behavioral attitudes toward the SARS-CoV-2 pandemic can theoretically be modeled using evolutionary game theory (with BIS and SB impacting on social decision-making in manifold ways). Game-theoretical paradigms create situations requiring individual decisions to cooperate or defect (at the cost of others). Most neuroeconomic games concern the distribution of quantifiable (often monetary) resources between two or more parties—individuals, groups or nations [16].

Concerning the topic discussed here, the Public Goods Game (PGG) may be most informative. As far as health issues are concerned, the PGG has previously been utilized to explain social dilemmata arising from the pros and cons of vaccination, herd immunity and use of antiviral drugs [17]; the PGG has, however, not been applied to the spread of infectious diseases, or the prevention thereof, when no vaccination is available.

The most common version of the PGG is played by an optional number of players who receive a defined amount of money or number of tokens at the beginning of the exchange scenario. Participants are asked to simultaneously invest their money in a common pool (the public good), usually without knowing the allowance of the other players. An experimenter multiplies the whole sum by a factor that is larger than one but smaller than the number of players, and returns an equal share of that money to each player. In other words, all players benefit equally, irrespective of how much they have invested before. If someone chooses a free-riding strategy while letting the others make their contributions, his or her return will exceed those of the other players [18, 19]. If played iteratively, investments usually decline over successive rounds, unless non-cooperative behavior can be sanctioned by the other players [20]. If punishment of selfish behavior becomes part of the game, investments increase significantly and remain stable across trials [21]. Interestingly, research utilizing the PGG has also demonstrated the occurrence of ‘antisocial punishment’, that is, the sanctioning of cooperative behavior [22]. Strangely enough, at first sight, some individuals tend to punish altruistic acts, particularly if the punishment can be performed anonymously. The motivations for antisocial punishment can be diverse, ranging from a desire to dominate others, competitiveness, or derogation of ‘do-gooders’. Conversely, antisocial punishment is constrained by strong norms of civic cooperation, but less well contained in societies with weaker norms, which is, in part, explained by cultural differences in collectivism versus individualism [22].

In contrast to social decision-making in relation to the distribution of quantifiable goods, the definition of ‘public health’ as a ‘public good’ has instilled controversy [23]. In fact, it has been argued that a public good comprises three elements: First, it must be a ‘good’; second, it is non-rivalrous (i.e. the consumption of the good by someone does not preclude others to benefit from it); third, it is non-excludable (i.e. one cannot be prevented to enjoy it). According to these criteria, herd immunity (an important goal in the handling of epidemics or pandemics, usually achieved through vaccination) constitutes a public good: first, it protects people from developing infectious diseases; second, immunization of a person does not preclude others from protection; and third, once herd immunity has been established, no one can be excluded from the protection it provides. Framed in a PGG, most people comply with mandatory vaccination regulations (thus contributing to the public good of herd immunity). As herd immunity is already achieved in vaccination programs, if only 90% of the population have been vaccinated, the public good of ‘herd immunity’ can even tolerate [17, 23].

However, as Dees (2018) argues, sanitation and clean water would not fall under this definition of a public good, because neither is non-rivalrous or non-excludable. Indeed, many people around the world are denied access to clean water and sanitation, and enjoying either one can be quite competitive. According to Dees (2018), public health as a public good thus warrants a normative component. Put another way, sanitation and clean water *ought* to be non-rivalrous and non-excludable to qualify as a ‘normative public good’.

This distinction is important for the understanding of how epidemics and pandemics are dealt with. A normative public good, as Dees [23] points out, has four elements: first, it is a ‘good’; second, everyone has unlimited access to it, that is, it is non-rivalrous and non-excludable; third, it benefits society (the public) through collective effort; fourth, the good is important enough to justify collective effort, a point that is open to debate, and thus also relevant for public health regulations in relation to the SARS-CoV-2 pandemic. In any event, by definition, combatting the SARS-CoV-2 pandemic qualifies as a normative public good in every sense.

With this evolutionary background, we may now re-interpret several social-behavioral aspects of the pandemic:

### Cooperation versus ‘defection’

In the absence of a vaccination against SARS-CoV-2, cooperation in this kind of PGG encompasses a variety of behavioral means to reduce the risk of virus transmission. These include ‘social distancing’, wearing face masks and identifying individuals who show signs suspect of infection. This is a mammoth task for society, because virtually everyone needs to comply to achieve the public good of slowing the spread of infection. While being far from perfect, these measures help slow down the number of new infections and protect those at increased risk of detrimental outcomes of COVID-19. Activation of BIS is probably supportive in this regard.

However, social distancing has predictable downsides. One is that social isolation is stressful and detrimental to health and immune function [9]. Moreover, social distancing does not work the same for individuals showing SB, and it may even not be required from those who have recovered from COVID-19. In addition, BIS activation may be associated with anxiety to spread more quickly as the virus itself. Indeed, it can be dangerous, if not fatal, to overlook someone who is infectious, but inexpensive to believe someone is infectious who, in fact, is not. Such hypervigilance can expand quickly in populations via social or vicarious learning [24]. Accordingly, the dispersal of threat-detection mechanisms faster than the infectious agent may give an additional survival advantage at the cost of false positives. It is little more than speculation that social prejudices may impact on the number of false positives, which would likely include socially disadvantaged people, racial minorities, and perhaps mentally ill [15].

In contrast to cooperation in this special PGG, defining ‘free-riding’ or ‘defection’ is much more difficult. Indeed, in neuroeconomic games, the failure to cooperate is usually conceived of as an intentional act. This is much less clear in a normative PGG where public health is the public good. More specifically, there is certainly a minority of individuals who deliberately defect, because they are willing to take the risk of own infection, and, at the same time, do not care about the health of others. The percentage of such antisocial behavior may be low, however, particularly when compared to those who inadvertently do not cooperate. Accordingly, inadvertent non-cooperation pertains to the very young, people with cognitive impairment insofar as it compromises rational decision-making, and asymptomatic virus carriers. In the words of PGG, these individuals would count as ‘defectors’, even in the absence of deliberate choice to not cooperate. With regard to a ‘normative public good’ such as public health, however, non-compliance with rules of social distancing cannot count as ‘free-riding’, because there is no short or long-term benefit for those who ‘defect’. Reinforcing cooperation by public health regulations for these social groups is thus possible only by incurring costs on third parties, which includes parents (of young children), caretakers of elderly (e.g. at home or in nursing homes) etc.

### Development over time

Theoretically, achieving herd immunity against SARS-CoV-2 slowly is, aside from the search for a vaccine, one potential strategy to cope with the pandemic. The strategy is, however, fraught with unpredictable consequences, particularly in terms of casualties and the risk for health services (and the economy) that a large proportion of the population could fall ill at the same time. Unlike vaccination campaigns aiming at herd immunity, however, the goal to slow down SARS-CoV-2 infection rates has a different temporal dynamic, in fact, an unpredictable dimension with respect to time.

This aspect is highly relevant, because the PGG logic predicts that cooperation decreases over time, particularly, if no sanctions have been put into place or, in the case of the pandemic, may not work for reasons outlined above. It is already observable in many countries around the world that, if political decision-makers lift public health regulations too early, infection rates increase rapidly (as well as the death toll of COVID-19). It is thus beyond fortune-telling to predict that almost certainly second and third infection waves will hit the globe.

### Antisocial punishment

Strangely enough, but also predictably from a game-theoretical point of view, one can also observe antisocial punishment in the current pandemic, at least indirectly and in subtle ways. This is a much more contentious issue compared to the points discussed above, because, as we will argue, antisocial punishment in relation to the SARS-CoV-2 pandemic comes in different disguises.

One is the frank denial of the existence of the infectious agent itself, or the view that the pandemic is much less dangerous as suggested by virologists and other medical experts. Denying or belittling medical concerns about the significance of the pandemic by official institutions including governments invites people to not obey to the rules of social distancing, perhaps more so those whose BIS is only mildly activated.

Another (indirect) way to undermine cooperation in this particular PGG is the spread of irrational or even delusional ideas about the origin of the virus or the idea that some socially high-ranking individual utilizes the pandemic to take control over the world. Such bizarre propositions, we believe, arise from ancestral fears, where invisible agents causing sickness and/or death incite suspiciousness against outgroups or putatively dangerous within-group coalitions [25]. That is, an enemy not from a competing group must come from one’s ingroup, usually someone who has great power or influence, or someone who lacks protection from kin (accusation of witchcraft, etc.) [26]. This is a particularly dangerous aspect of the current crisis, because modern technology accelerates the distribution of misinformation without effective control of its veracity. From a psychiatric point of view, the distinction between conspiracy theories and delusional ideation may become reduced, idiosyncratic or volatile.

In any event, conspiracy theories erode collective efforts to control the spread of the virus, and they can be specifically detrimental to the most vulnerable parts of society, i.e. at-risk subjects or individuals presenting with SB. This is particularly the case, if conspiracy theories include the notion that vaccination against SARS-CoV-2 (once available) may be come at the cost of being ‘tagged’. Even if we do not know at present, whether or not vaccination will become available, and how effectively it will protect against COVID-19, the prospect that a sizeable number of individuals may fall prey to conspiracy theories and thus refuse vaccination is grim. Textbox 1 summarizes the most important insights and predictions from evolutionary theory on the SARS-CoV-2 pandemic.

## SUMMARY

The coronavirus crisis has created a natural experiment that has put ancestral means of controlling the spread of contagious disease in small-scale communities to the test in contemporary mass societies. Individuals greatly differ with regard to their susceptibility to COVID-19 with a great many asymptomatic or only mildly ill, yet others with severe syndromes that have a wide range of dramatic organic disease. Although research about the genetics underlying individual differences in vulnerability is still in its infancy, the human leukocyte antigen (HLA) may play a critical role [27]. In addition, there is limited evidence to suggest that people with blood group A carry a greater risk for detrimental outcomes of COVID-19 as opposed to 0 [28]. Recent research indicates, however, that it is possibly not the blood group *per se* that confers risk for COVID-associated pneumonia. Instead, the presence of Anti-A antibodies seems to be protective against severe lung affection, particularly IgG [29].

Social contact has been crucial to fitness over millions of years in our evolutionary history, and failure to achieve social connection can therefore activate stress mechanisms that realize immediate survival at the expense of longer-term health. As West-Eberhard put it, ‘individuals of social species having these specialized characteristics are in a sense trapped into group life, and group living may become virtually ‘obligatory’ for them.’ [30, p. 224]. Yet, physical distancing is not necessarily equal to social distancing, particularly not in our species, as language and perhaps laughter evolved to allow ‘social grooming’ at a distance, with remarkable physical effects mediated by endogenous neuropeptides that are good for positive mood and thus mental health [31].

The pandemic will remain a challenge at least for an indeterminate and perhaps lengthy time. Health care systems in developed countries erroneously believed that communicable diseases were primarily a threat to the less developed part of the planet, only to learn that the systems are vulnerable to unexpected (though predicted) attacks from the viral world [32]. As the virus continues to evolve during its propagation around the world, there is hope it may become more contagious, but less fatal (reduced virulence), though this is at present uncertain [33, 34]. Unlike previous pandemics or endemic events, this pandemic will most likely leave no selective mark on the gene pool, because the vast majority of fatalities occur beyond the reproductive lifespan. With the first measures taken by governments to lift the shut-down, it needs to be kept in mind that decisions over the reduction of the burden of ‘social distancing’ are largely driven by economic policy, not medical advice. Public health authorities need to take action to reduce incipient or expressed policies of Social Darwinism. Textbox 2 comprises suggestions for public health measures derived from evolutionary insights.

Moreover, in the aftermath of the pandemic there likely will be a second epidemic of people who are grieving because they lost loved-ones to the pandemic, or people suffering from posttraumatic stress disorder (notably among hospital staff). Individuals may develop depression, anxiety disorders, addiction or other maladaptive stress-coping conditions. This may pose a challenge specifically on mental health workers and psychiatrists. In any event, the Coronavirus crisis may be taken as a chance to prepare for even more dangerous pandemics that will certainly besiege an ever-increasing globalized world.

*Conflict of interest*. None declared.

**Textbox 1. Bullet points derived from evolutionary insights about the SARS-CoV-2 pandemic**

- In times of pandemics, people activate ancestral behavioral mechanisms to reduce the risk of infection (i.e. BIS), also impacting social life.
- Game-theoretical approaches suggest that a significant (and as the pandemic continues) and growing number of people with fail to comply with social rules to keep infection rates low.
- Accordingly, almost certainly second and third waves of COVID-19 will occur, unless an efficient vaccine will be available on a mass scale.
- Antisocial punishment poses a threat to public health, as it undermines efforts to control the disease.

**Textbox 2. Suggestions for public health measures**

- The most relevant issue is continuing medical education of the public about SARS-CoV-2 to minimize the risk of defection and the spread of false information.
- Regulation of social distancing and other measures that prevent virus transmission (e.g. compulsory face masks) is imperative. A lesson learnt from game theory is that this must entail punishment for non-compliance.
- Preparation for a second (and third) wave is warranted, as well as for secondary health issues that may affect vulnerable or at-risk populations most, including psychological problems following prolonged social isolation.
